# Development of a School-Based Online Periodontal Education Programme for Adolescents

**DOI:** 10.1016/j.identj.2024.07.002

**Published:** 2024-07-23

**Authors:** Satoru Haresaku, Akiko Chishaki, Junko Hatakeyama, Yasunori Yoshinaga, Junko Yoshizumi, Mito Yamamoto, Etsuko Matsuzaki, Ippei Hamanaka, Takashi Tsutsumi, Yusuke Taniguchi, Kimiko Ohgi, Masahiro Yoneda

**Affiliations:** a Department of Nursing, Fukuoka Nursing College, Fukuoka, Japan; b Medical Examination Center, Fukuoka Dental College, Medical and Dental General Hospital, Fukuoka, Japan; c Section of General Dentistry, Department of General Dentistry, Fukuoka Dental College, Fukuoka, Japan; d Section of Periodontology, Department of Odontology, Fukuoka Dental College, Fukuoka, Japan; e Oral Medicine Research Centre, Fukuoka Dental College, Fukuoka, Japan; f Section of Oral Oncology, Department of Oral & Maxillofacial Surgery, Fukuoka Dental College, Fukuoka, Japan; g Fukuoka Dental Hygienist School, Fukuoka, Japan; h Section of Operative Dentistry and Endodontology, Department of Odontology, Fukuoka Dental College, Fukuoka, Japan; i Section of Removable Prosthodontics, Department of Oral Rehabilitation, Fukuoka Dental College, Fukuoka, Japan; j The Center for Visiting Dental Service, Department of General Dentistry, Fukuoka Dental College, Fukuoka, Japan; k Section of Oral Implantology, Department of Oral Rehabilitation, Fukuoka Dental College, Fukuoka, Japan

**Keywords:** Online oral health education, E-learning, Periodontal disease, Gingivitis, Adolescent

## Abstract

**Introduction and aims:**

This study aimed to investigate the effects of a developed school-based online health education programme with a periodontal examination results sheet for high school students on their subjective oral symptoms, knowledge and attitudes regarding oral health, and oral health behaviours.

**Methods:**

The participants were first- and second-year students aged 15 to 17 years (n = 847) at a high school in Japan. The students underwent a periodontal examination and were divided into periodontal condition (PC) and nonperiodontal condition (non-PC) groups. The students participated in the online oral health education programme, which included a periodontal examination results sheet after the examination. The data for identifying the effect of the programme were collected via questionnaire surveys at the periodontal examination (baseline), after 3 months, and after 1 year, and they were compared between baseline and 3 months later and between baseline and 1 year later by the chi-square test. Logistic regression analysis was used to determine the associations between the measured variables related to oral health at 3 months or 1 year and the presence/absence of periodontal conditions after adjustment for sex and variables at baseline.

**Results:**

A total of 628 students (74.1%) participated in this study. The percentages of individuals with knowledge of how many teeth they had, knowledge of periodontal disease, and experience with toothbrushing instruction increased significantly after 1 year in both groups (*p* < .05). The awareness of gingival swelling and bleeding and the use of fluoride toothpaste at 3 months were positively associated with the presence of periodontal conditions.

**Conclusions:**

This study showed that an online oral health education programme contributed to improving oral health knowledge and behaviours among high school students and that the awareness of periodontal conditions according to the periodontal examination results sheet might improve the awareness of gingival swelling and bleeding at 3 months.

## Introduction

Periodontal disease is widespread, with prevalence rates ranging from 2.3% to 33.5%,^1^ and approximately 19% of people aged 15 years and older have severe periodontal disease, with more than 1 billion patients worldwide.^2^ Periodontal disease leads to tooth loss^3^ and can lead to systemic diseases such as diabetes^4^ and cardiovascular disease.^5^

Gingivitis has also been reported to be associated with systemic diseases such as otitis media and asthma in adolescents.^6^ Furthermore, persistent gingival inflammation in adolescents is associated with periodontitis and tooth loss in adulthood.^7^ Therefore, the implementation of dental health education programmes to improve periodontal disease status in adolescents is important for preventing gingivitis in adolescents and preventing periodontitis and tooth loss in adulthood.

Schools provide an ideal setting for delivering equally oral health education in combination with preventive services to achieve oral health promotion.^8^ Regardless of the family's socioeconomic status, students can receive the benefits of the intervention.

School-aged adolescents in particular are in need of preventive programmes to ensure positive long-term dental health and hygiene.^9^^,^^10^ Previous studies have reported the effectiveness of school-based oral health education intervention programmes for improving adolescents’ oral hygiene or gingival health.^11^, ^12^, ^13^, ^14^, ^15^, ^16^, ^17^ In addition, a few previous studies have reported the effectiveness of online-based oral health education for schoolchildren aged 10-11 years and adults.^18^^,^^19^ However, to our knowledge, no studies have reported the effectiveness of school-based online oral health education for adolescents.

In Japan, school health activities such as dental examinations and examination result notifications for pre-, elementary, junior high, and high school students are mandated by the School Health Law.^20^ However, the prevalence of periodontal disease (≥4-mm pocket depth) among adolescents aged 15 to 19 years was 17.8% in 2022,^21^ increasing with age and exceeding 50% from age 65 years. In addition, the prevalence of periodontal disease among individuals aged 15 to 24 years increased from 17.6% in 2016 to 17.8% in 2022, although the decayed, missing and filled teeth (DMFT) index in individuals aged 15 to 24 years decreased from 3.5 in 2016 to 2.5 in 2022.^21^

A recent systematic review of school dental screenings reported that the effectiveness of traditional screenings for improving oral health status is uncertain compared to that of no screening.^22^ Therefore, the increase in periodontal disease among adolescents in Japan may suggest the need to develop effective school-based oral health education programmes to prevent periodontal disease.

After the coronavirus disease 2019 (COVID-19) pandemic, the use of online learning spread rapidly and was implemented in various educational institutions in Japan.^23^ One of the advantages of online education compared to traditional face-to-face education in each classroom is that an oral health educator can teach all students simultaneously throughout the school from a single delivery site, which would mean a reduction in cost and implementation burden. Therefore, a programme that combines online oral health education programmes focusing on periodontal disease with the distribution of periodontal disease examination results was developed by a Japanese dental examination and research team, and the programme was implemented for high school students.

This study aimed to investigate the effects of the developed school-based online oral health education programme for high school students on their subjective oral symptoms, oral health knowledge and attitudes, and oral health behaviours.

## Methods

### Study population

The study participants consisted of first- and second-year students (n = 847) aged 15 to 17 years at a high school in Fukuoka Prefecture, southwestern Japan. The school had a contract with the Medical Examination Centre, Fukuoka Dental College Medical and Dental General Hospital, to conduct research and oral health activities in 2022. A programme for high school students that combined online oral health education focused on periodontal disease with a periodontal disease examination results sheet was developed by the dental examination and research team at the centre and was implemented for the participants.

In the first phase of the programme, a baseline questionnaire survey and periodontal examination were administered to the students (Figure). The questionnaires were distributed by classroom teachers from 10 to 17 October 2022. The consent form was the first page of the questionnaire. The students were informed about this research by the schoolteachers, asked to sign the consent form if they agreed to participate, and the questionnaire was administered during their periodontal examination. The periodontal examination was conducted on 17 November 2022 in the school hall. In the second phase, the first and second online oral health education interventions were implemented for the students by Zoom (Zoom Video Communications Corporation, San Jose, CA, USA) on 13 December 2022 and 16 January 2023, respectively. The periodontal examination results sheet was distributed to the students during the second intervention. The second and third questionnaire surveys were administered from 23 to 30 January 2023 and from 27 September to October 2 2023, respectively. The participants were divided into a periodontal condition (PC) or nonperiodontal condition (non-PC) group according to the presence or absence of periodontitis on the distributed examination results sheet, as differences in periodontal condition may affect the effectiveness of the programmes. The subjective oral symptoms, oral health knowledge and attitudes, and oral health behaviours of each group at baseline, 3 months, and 1 year were assessed via questionnaire surveys. The students who participated in all the questionnaire surveys were included in the analysis.

**Figure:**
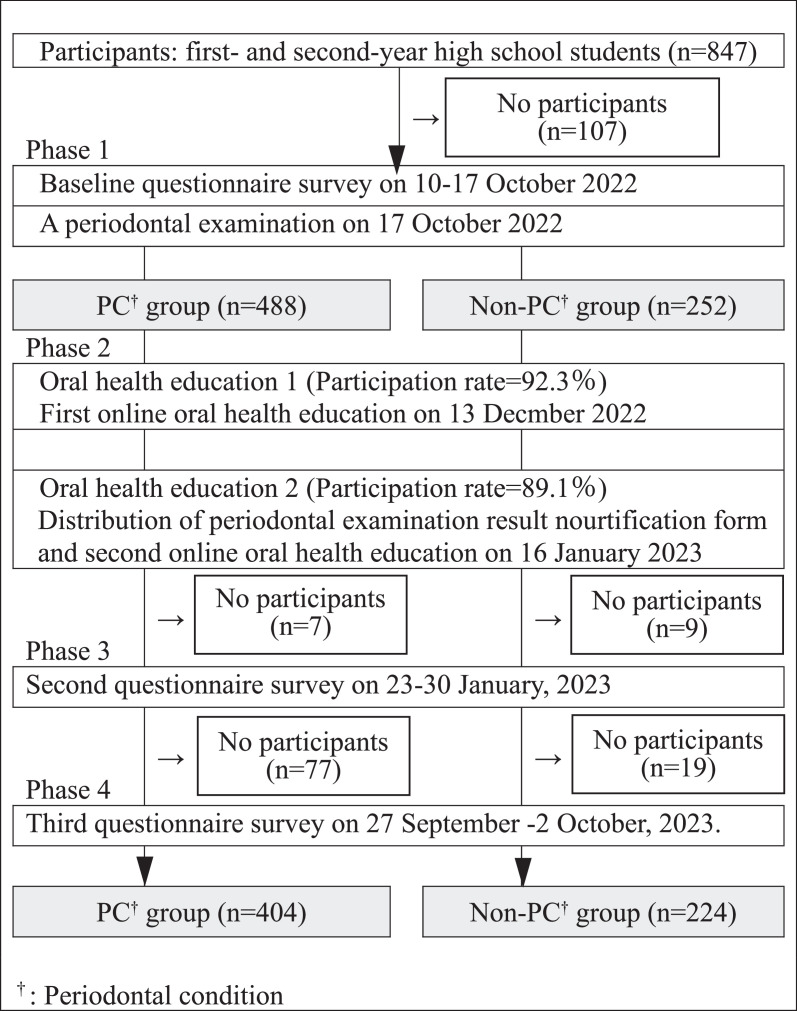
Flow chart of this study.

### Ethical considerations

This study was conducted in accordance with the ethical standards outlined in the 1964 Declaration of Helsinki and received ethical approval from the Ethics Committee of Fukuoka Gakuen, Fukuoka, Japan (approval no. 548). Written informed consent was obtained from all participants prior to their participation in this study.

### Clinical examination

A periodontal examination (partial community periodontal index (PCPI)^24^) was conducted by 10 dentists using a WHO probe and a dental mirror with LED light (BSA Sakurai Ltd.). The PCPI was used to examine the periodontal condition (gingival bleeding, calculus, and pocket depth) of 6 permanent teeth (16, 11, 26, 36, 31, and 46) based on the probing pocket depth at 6 sites around the teeth. The second molars that were examined via a standard PCPI were excluded from this study because there were some cases in which the second molars had not erupted.

The periodontal conditions of healthy, bleeding, calculus, a pocket depth of 4 to 5 mm, and pocket depth of 6 mm or more were coded as 0, 1, 2, 3, and 4, respectively. The participants in the PC group had codes 1 to 4 for any of the examined teeth.

### Questionnaire

The questionnaire was based on a previously developed questionnaire that is normally used in the Japan Academy of Dental Human Dock.^25^

The questionnaire consisted of demographics and 3 categories: subjective oral symptoms, oral health perceptions, and oral health behaviours (Supplementary Table 1). The demographics were sex and school year. First-year students were aged 15 to 16 years, and second-year students were aged 16 to 17 years.

The questions about subjective oral symptoms (4 items), perceptions of oral health (5 items), and oral health behaviours (9 items) were extracted from a previously developed questionnaire^25^ and limited to items that seemed to be related to periodontal disease. The answers to the questions about subjective oral symptoms and perceptions of oral health were all ‘Yes’ or ‘No’. Regarding oral health behaviours, the answer choices were ‘3 times or more’, ‘2 times’, and ‘1 time or less’ for the question about how many times the teeth were brushed per day; ‘5 minutes or more’, ‘3 to 5 minutes’, and ‘less than 3 minutes’ for the question about how long the students brushed their teeth for each time; and ‘Yes’ and ‘No’ for the other questions (Supplementary Table 1).

The answer choices of ‘Yes’, ‘3 times or more’, and ‘5 minutes or more’ in questions except for questions regarding negative attitudes towards oral health (fear of dental treatments) and bad oral health behaviours (drinking sports drinks frequently and eating many sweet foods) were coded as ‘1’, and answers of ‘No’, ‘2 times or less’, and ‘less than 5 minutes’ were coded as ‘0’. The answer choices of ‘Yes’ in the questions regarding the negative attitudes and bad oral health behaviours were coded as ‘0’, and answers of ‘No’ were coded as ‘1’.

The total replaced number in each category was summed as the level of subjective oral symptoms (levels 0-4), good knowledge and attitudes regarding oral health (levels 0-5), and good oral health behaviours (levels 0-11).

### Online oral health education programmes

The programme consisted of a combination of videos on oral health and live videos of oral health instruction provided by a dental hygienist (Supplementary Table 2). The videos regarding oral health were made by oral health professional members of a working group to promote oral health and to increase interest in entering oral health professional schools for high school students, supported by the Japanese Ministry of Education, Culture, Sports, Science and Technology. The videos consisted of 4 chapters, “Chapter 1: Sports dentistry”, “Chapter 2: Introduction of Oral Health Professionals”, “Chapter 3: Periodontal Disease”, and “Chapter 4: Oral Hygiene Methods”. The first educational programme consisted of video delivery of Chapters 1 and 2 and live video delivery of the commentary of the chapters by a dental hygienist. Before the second educational programme, the periodontal disease examination results sheet and toothbrush were distributed to each student, and a dental hygienist gave a lecture on how to read the sheet with a ten-minute PowerPoint after the video distribution of Chapter 3. The dental hygienist gave instructions on how to brush teeth with a dental care model, a toothbrush and dental floss for 15 minutes after the video distribution of Chapter 4.

### Periodontal examination results sheet

The periodontal examination results sheets distributed to the participants are shown in Supplementary Figure 1. The sheet was made and printed using FileMaker Pro 14 (Version 14.01; Claris International Corporation). The layout of the sheet consisted of periodontal examination results columns at the top of the sheet and oral health information at the bottom. In the periodontal examination results column, “Presence” or “Absence” was added to the “Bleeding” and “Tartar” columns, and “Moderate,” “Severe,” or “Absence” was added to the “Swollen gum” columns in each sextant.

The oral health information section on the sheet included explanations about gingivitis and calculus, the systemic effects of periodontal disease, methods of preventing periodontal disease, and the benefits of regular dental visits because those oral health behaviours were significantly associated with periodontal disease findings in a previous study conducted among the same participants in this study,^26^ and they were considered important for improving their periodontal condition.

### Statistical analyses

The chi-square test was used to compare differences in subjective oral symptoms, knowledge and attitudes regarding oral health and oral health behaviours between baseline and 3 months later and between baseline and 1 year later. The Friedman's test was used to compare the levels of subjective oral symptoms, good knowledge and attitudes regarding oral health, and good oral health behaviour among the survey periods: baseline, 3 months, and 1 year. To investigate the effect of the periodontal examination results sheet on subjective oral symptoms, knowledge and attitudes regarding oral health and oral health behaviours among the participants, the associations between the variables measured at 3 months or 1 year and the periodontal examination results at baseline (the presence/absence of a periodontal condition) were analysed. A chi-square test was used to compare differences in variables related to oral health between patients with and without a periodontal condition at each time point. Then, logistic regression analysis was used to investigate the associations between the variables measured at 3 months or 1 year and the presence/absence of a periodontal condition after adjustment for sex and each of the variables at baseline. The dependent variables were the variables related to oral health (replacing the answer choices for each question with ‘0’ or ‘1’) measured at 3 months or 1 year. The independent variables were periodontal conditions (presence=1 and absence=0), sex (male=1 and female=0), and each of the variables measured at baseline (replaced numbers with the numbers ‘0’ or ‘1’).

The data were analysed with a 5% significance level. The statistical analyses were performed using IBM SPSS Statistics software (version 21.0; IBM Corporation).

## Results

A total of 740 students (359 males and 381 females) participated in the first questionnaire survey and periodontal examination (Figure 1), and they were divided into a PC group (n = 488) and a non-PC group (n = 252). A total of 628 (74.1%) students, 224 in the PC group and 404 in the non-PC group, responded to the questionnaire survey. The number (%) of participants was 782 (92.3%) in the first programme and 755 (89.1%) in the second programme.

### Changes in subjective oral symptoms

Overall, 7.3% of the students experienced gingival swelling at baseline, which significantly increased to 10.8% after 3 months (*p* = .031) but decreased to 8.6% after 1 year (Table 1 and Supplementary Figure 2). Approximately 20% of the students experienced gingival bleeding, toothache, or bad breath, and the percentages did not change significantly after 1 year. The change in the awareness of gingival swelling in the PC group was similar to the change in the total group, with a significant difference in the awareness of gingival swelling between baseline and 3 months later (*p* = .003), but no significant difference between baseline and 1 year later. In the non-PC group, 17.0% of the students experienced gingival bleeding; this percentage decreased significantly to 9.4% 3 months later but increased to 12.9% 1 year later, and there was no significant difference in the awareness of gingival bleeding between baseline and 3 months later or between baseline and 1 year later.

**Table 1:** Changes in subjective oral symptoms, knowledge and attitudes regarding oral health, and oral health behaviour in the PC† and non-PC† group.

	Total					PC† group (n = 404)					Non-PC* group (n = 224)					
	Baseline n (%)	3-month n (%)	1-year n (%)	*p* Value†	*p* Value‡	Baseline n (%)	3-month n (%)	1-year n (%)	*p* Value†	*p* Value‡	Baseline n (%)	3-month n (%)	1-year n (%)	*p* Value†		*p* Value‡
Subjective oral symptom																
Gingival swelling	46 (7.3)	68 (10.8)	54 (8.6)	.031	.409	31 (7.7)	58 (14.4)	39 (9.7)	.003	.322	15 (6.7)	10 (4.5)	15 (6.7)	.303		1.000
Gingival bleeding	119 (19.0)	107 (17.0)	97 (15.4)	.364	.095	81 (20.1)	86 (21.3)	68 (16.8)	.690	.225	38 (17.0)	21 (9.4)	29 (12.9)	.018		.233
Toothache	124 (19.8)	134 (21.4)	105 (16.7)	.494	.157	80 (19.9)	91 (22.6)	67 (16.6)	.343	.229	44 (19.7)	43 (19.2)	38 (17.0)	.887		.450
Bad breath	148 (23.7)	135 (21.5)	131 (20.9)	.355	.230	104 (25.9)	95 (23.5)	88 (21.8)	.426	.167	44 (19.6)	40 (17.9)	43 (19.2)	.628		.905

Knowledge and awareness regarding oral health																

Knowledge of how many teeth they have	48 (7.6)	135 (21.5)	97 (15.4)	<.001	<.001	26 (6.4)	81 (20.0)	60 (14.9)	<.001	<.001	22 (9.8)	54 (24.1)	37 (16.5)	<.001	.036	
Fear of dental treatments	205 (32.6)	195 (31.2)	192 (30.6)	.571	.441	143 (35.4)	130 (32.2)	123 (30.4)	.334	.134	62 (27.7)	65 (29.3)	69 (30.9)	.708	.449	
Interest in oral health	235 (37.5)	255 (40.7)	279 (44.4)	.247	.012	150 (37.2)	167 (41.3)	179 (44.3)	.231	.041	85 (37.9)	88 (39.5)	100 (44.6)	.742	.150	
Knowledge of periodontal disease	338 (53.9)	499 (79.5)	430 (68.8)	<.001	<.001	219 (54.3)	321 (79.5)	278 (69.0)	<.001	<.001	119 (53.1)	178 (79.5)	152 (68.5)	<.001	.001	
Knowledge of the effect of oral diseases on systematic diseases	411 (65.6)	483 (76.9)	482 (76.8)	<.001	<.001	268 (66.3)	310 (76.7)	306 (75.7)	.001	.003	143 (64.1)	173 (77.2)	176 (78.6)	.002	.001	

Oral health behaviour																

Frequency of toothbrushing per day (≧3 times)	64 (10.2)	76 (12.1)	69 (11.0)	.282	.647	36 (8.9)	43 (10.6)	41 (10.1)	.407	.549	28 (12.5)	33 (14.7)	28 (12.5)	.491	1.000	
Drinking sports drinks frequently	98 (15.6)	61 (9.7)	73 (11.6)	.002	.039	71 (17.6)	45 (11.1)	47 (11.6)	.009	.017	27 (12.1)	16 (7.1)	26 (11.6)	.075	.870	
Dental floss use	173 (27.6)	202 (32.2)	179 (28.5)	.077	.719	96 (23.8)	117 (29.0)	98 (24.3)	.098	.885	77 (34.4)	85 (37.9)	81 (36.2)	.431	.692	
Time for toothbrushing per brushing (≧5 minutes)	299 (47.6)	389 (61.9)	344 (54.8)	<.001	.011	186 (46.0)	242 (59.9)	212 (52.5)	<.001	.067	113 (50.4)	147 (65.6)	132 (58.9)	.001	.071	
Visiting dentists regularly (more than once a year)	312 (49.8)	330 (52.9)	337 (53.7)	.269	.158	184 (45.5)	197 (49.0)	210 (52.0)	.325	.067	128 (57.4)	133 (59.9)	127 (57.0)	.591	.924	
Fluoride toothpaste use	318 (50.8)	390 (62.2)	390 (62.2)	<.001	<.001	196 (48.6)	257 (63.8)	249 (61.8)	<.001	<.001	122 (54.7)	133 (59.4)	141 (62.9)	.319	.077	
Eating many sweet foods	419 (66.8)	395 (62.9)	397 (63.2)	.145	.180	260 (64.4)	245 (60.6)	246 (60.9)	.276	.309	159 (71.3)	150 (67.0)	151 (67.4)	.321	.372	
Experience with toothbrushing instruction	454 (72.3)	505 (80.4)	534 (85.0)	.001	<.001	280 (69.3)	323 (80.0)	338 (83.7)	.001	<.001	174 (77.7)	182 (81.3)	196 (87.5)	.349	.006	
Toothpaste use	495 (79.1)	506 (80.6)	512 (81.7)	.508	.249	317 (78.7)	328 (81.2)	332 (82.2)	.370	.208	178 (79.8)	178 (79.5)	180 (80.7)	.925	.812	

⁎ Periodontal condition.

† Comparison at baseline and 3 months later, chi-square test.

‡ Comparison at baseline and 1 year later, chi-square test.

### Changes in knowledge and attitudes regarding oral health

The percentages of participants who were aware of how many teeth they had, of periodontal diseases, and of the effect of oral diseases on systemic disease at baseline were 7.6%, 53.9%, and 65.6%, respectively (Table 1 and Supplementary Figure 3). And these percentages increased significantly 1 year later (*p* < .001). By group, increases in the percentages of these knowledge were also found in both the PC and non-PC groups (*p* < .05). The percentage of participants who were interested in oral health at baseline was 37.5%, which increased significantly to 44.4% after 1 year (*p* = .012). By group, the percentage of interest in oral health among those in the PC group increased significantly from 37.2% at baseline to 44.3% 1 year later (*p* = .041).

### Changes in oral health behaviours

Overall, the percentages of patients who brushed their teeth more than 5 minutes, used fluoride toothpaste, and had received toothbrushing instruction at baseline were 47.6%, 50.8%, and 72.3%, respectively (Table 1 and Supplementary Figure 4). And these percentages increased significantly to 54.8%, 62.2%, and 85.0%, respectively, 1 year later (*p* < .05). In contrast, 15.6% of the participants drank sports drinks frequently at baseline, but this percentage decreased significantly to 11.6% 1 year later. The percentages of other oral health behaviours did not change significantly 1 year later. By group, the percentage of patients who brushed their teeth more than 5 minutes each time significantly increased 3 months later (*p* < .001) but not 1 year later in both groups. The percentages of patients who drank sports drinks frequently and used fluoride toothpaste improved significantly 1 year later in only the PC group (*p* < .05).

### Changes in the levels of subjective oral symptoms, good knowledge and attitudes regarding oral health, and good oral health behaviour

The lowest mean level (standard deviation: SD) of subjective oral symptoms in the PC group was 0.62 (0.88) at 1 year, and that of the non-PC group was 0.51 (0.77) at 3 months (Table 2). There were significant differences in the levels of subjective oral symptoms among the survey periods in the total group and the PC group but not in the non-PC group.

**Table 2:** Changes in levels of subjective oral symptom, good knowledge and attitudes regarding oral health, and good oral health behaviour in the PC* and non-PC* group.

		Baseline	3-month	1-year	
	Group	Mean level (SD†)	Mean level (SD†)	Mean level (SD†)	*p* value║
Level of subjective oral symptom (0-4) ‡	Total	0.68 (0.88)	0.71 (0.90)	0.62 (0.88)	.035
	PC*	0.72 (0.88)	0.81 (0.94)	0.65 (0.89)	.001
	Non-PC*	0.63 (0.88)	0.51 (0.77)	0.56 (0.85)	.112
Level of good knowledge and attitude regarding oral health (0-5)§	Total	2.31 (1.10)	2.87 (1.16)	2.74 (1.15)	<.001
	PC*	2.28 (1.09)	2.85 (1.14)	2.73 (1.15)	<.001
	Non-PC*	2.37 (1.13)	2.90 (1.20)	2.75 (1.14)	.001
Level of good oral health behaviour (0-11)¶	Total	4.53 (1.53)	5.08 (1.53)	5.01 (1.47)	<.001
	PC*	4.38 (1.49)	5.00 (1.57)	4.94 (1.49)	<.001
	Non-PC*	4.81 (1.55)	5.23 (1.44)	5.15 (1.43)	<.001

⁎ Periodontal condition.

† Standard deviation.

‡ Higher scores indicated more subjective oral symptoms.

§ Higher scores indicated better knowledge and attitudes regarding oral health.

¶ Higher scores indicated better oral health behaviours.

║ Friedman's test.

The highest mean levels of good knowledge and attitudes were observed at 3 months in both the PC and non-PC groups, and the mean levels (SD) were 2.85 (1.14) in the PC group and 2.90 (1.20) in the non-PC group. The highest mean levels of good oral health behaviour were observed at 3 months in both groups, and the mean levels (SD) were 5.00 (1.57) in the PC group and 5.23 (1.44) in the non-PC group. Significant differences in oral health knowledge and attitudes and oral health behaviours were found among the survey periods in both groups.

### Differences in subjective oral symptoms, good knowledge and attitudes regarding oral health and good oral health behaviours between participants with and without periodontal disease

There were significant differences in awareness of gingival swelling and bleeding at 3 months between the PC and non-PC groups, although there were no differences between the 2 groups at baseline or at 1 year (Table 3). There were significant differences in the use of dental floss at all time points, visiting dentists regularly at baseline and 3 months, fear of dental treatment and experience toothbrushing instructions at baseline between the PC and non-PC groups.

**Table 3:** Differences in subjective oral symptoms, good knowledge and attitudes regarding oral health, and good oral health behaviours in the PC* and non-PC* group at baseline, 3 months, and 1 year.

	Baseline			3-month			1-year		
Variable [Yes=1, No (reference)=0]	PC* group n (%)	Non-PC* group n (%)	*p* value‡	PC* group n (%)	Non-PC* group n (%)	*p* value‡	PC* group n (%)	Non-PC* group n (%)	*p* Value‡
Subjective oral symptom									
Having gingival swelling	15 (6.7)	31 (7.7)	.647	58 (14.4)	10 (4.5)	<.001	39 (9.7)	15 (6.7)	.205
Having gingival bleeding	81 (20.1)	38 (17.0)	.330	86 (21.3)	21 (9.4)	<.001	68 (16.8)	29 (12.9)	.197
Having toothache	80 (19.9)	44 (19.7)	.971	91 (22.6)	43 (19.2)	.322	67 (16.6)	38 (17.0)	.903
Having bad breath	104 (25.9)	44 (19.6)	.076	95 (23.5)	40 (17.9)	.098	88 (21.8)	43 (19.2)	.445

Good knowledge and attitude regarding oral health									

Having knowledge of how many teeth they have	26 (6.4)	22 (9.8)	.126	81 (20.0)	54 (24.1)	.236	60 (14.9)	37 (16.5)	.580
Being afraid of dental treatments†	143 (35.4)	62 (27.7)	.048	130 (32.2)	67 (29.9)	.557	123 (30.4)	70 (31.3)	.834
Having interest in oral health	150 (37.2)	85 (37.9)	.857	167 (41.3)	88 (39.5)	.647	179 (44.3)	100 (44.6)	.935
Having knowledge of periodontal disease	219 (54.3)	119 (53.1)	.769	321 (79.5)	178 (79.5)	.998	278 (69.0)	152 (68.5)	.894
Having knowledge of the effect of oral diseases on systematic diseases	268 (66.3)	143 (64.1)	.577	310 (76.7)	173 (77.2)	.887	306 (75.7)	176 (78.6)	.421

Good oral health behaviour									

Toothbrushing more than 3 times per day	36 (8.9)	28 (12.5)	.154	43 (10.6)	33 (14.7)	.132	41 (10.1)	28 (12.5)	.367
Drinking sports drinks frequently†	71 (17.6)	28 (12.5)	.095	45 (11.1)	16 (7.1)	.105	47 (11.6)	26 (11.6)	.992
Using dental floss	96 (23.8)	77 (34.4)	.005	117 (29.0)	85 (37.9)	.021	98 (24.3)	81 (36.2)	.002
Toothbrushing more than 5 minutes per brushing	186 (46.0)	113 (50.4)	.289	242 (59.9)	147 (65.6)	.157	212 (52.5)	132 (58.9)	.120
Visiting dentists regularly more than once a year	184 (45.5)	128 (57.4)	.004	197 (49.0)	133 (59.9)	.009	210 (52.0)	127 (57.0)	.232
Using fluoride toothpaste	196 (48.6)	122 (54.7)	.146	257 (63.8)	133 (59.4)	.277	249 (61.8)	141 (62.9)	.774
Eating many sweet foods†	260 (64.4)	160 (71.4)	.071	245 (60.6)	150 (67.0)	.116	246 (60.9)	151 (67.4)	.105
Experiencing with toothbrushing instruction	280 (69.3)	174 (77.7)	.025	323 (80.0)	182 (81.3)	.694	338 (83.7)	196 (87.5)	.197
Using toothpaste	317 (78.7)	178 (79.8)	.732	328 (81.2)	178 (79.5)	.601	332 (82.2)	180 (80.7)	.651

⁎ Non periodontal condition = 0, With periodontal condition = 1.

† Negative and reverse designed items.

‡ Chi-square test.

### Associations between variables related to oral health at 3 months or 1 year and the presence/absence of periodontal disease

Awareness of gingival swelling and bleeding at 3 months was positively associated with the presence of a periodontal condition, and the use of dental floss at 3 months and 1 year was negatively associated with the presence of a periodontal condition according to the univariate analysis (Table 4). After adjustment for sex and the variables at baseline, awareness of gingival swelling and bleeding and the use of fluoride toothpaste at 3 months were positively associated with the presence of periodontal conditions. The adjusted odds ratios (95% Confidence Interval: CI) of having awareness of gingival swelling, having awareness of gingival bleeding, and using fluoride toothpaste at 3 months for those with periodontal conditions were 4.38 (2.05-9.40), 3.03 (1.73-5.31), and 1.61 (1.08-2.42), respectively.

**Table 4:** Association between variables regarding oral health measured at 3 months or 1 year and the presence/absence of a periodontal condition* after adjustment for sex and each of the variables measured at baseline.

	3-month				1-year			
	Univariable analysis		Multivariate analysis		Univariable analysis		Multivariate analysis	
Variable [Yes=1, No (reference)=0]	OR (95% CI†)	*p* value¶	Adjusted OR‡ (95% CI†)	*p* value¶	OR (95% CI†)	*p* value¶	Adjusted OR‡ (95% CI†)	*p* value¶
Subjective oral symptom								
Having gingival swelling	3.59 (1.79-7.17)	<.001	4.38 (2.05-9.40)	<.001	1.49 (0.80-2.77)	.208	1.59 (0.77-3.30)	.212
Having gingival bleeding	2.61 (1.57-4.35)	<.001	3.03 (1.73-5.31)	<.001	1.36 (0.85-2.18)	.198	1.34 (0.78-2.28)	.285
Having toothache	1.23 (0.82-1.84)	.322	1.38 (0.85-2.26)	.194	0.97 (0.63-1.51)	.903	0.95 (0.59-1.53)	.829
Having bad breath	1.41 (0.94-2.14)	.099	1.23 (0.73-2.07)	.440	1.17 (0.78-1.76)	.445	0.90 (0.54-1.52)	.701

Good knowledge and attitude regarding oral health								

Having knowledge of how many teeth they have	0.79 (0.53-1.17)	.236	0.82 (0.53-1.28)	.388	0.88 (0.56-1.38)	.580	1.08 (0.64-1.83)	.776
Being afraid of dental treatments§	0.90 (0.63-1.28)	.558	1.18 (0.74-1.87)	.483	1.04 (0.73-1.48)	.834	1.39 (0.90-2.15)	.135
Having interest in oral health	1.08 (0.77-1.51)	.647	1.19 (0.81-1.76)	.375	0.99 (0.71-1.37)	.935	1.00 (0.70-1.44)	.993
Having knowledge of periodontal disease	1.00 (0.67-1.50)	.998	1.06 (0.69-1.64)	.776	1.02 (0.72-1.46)	.894	1.09 (0.74-1.60)	.651
Having knowledge of the effect of oral diseases on systematic diseases	0.97 (0.66-1.43)	.887	0.94 (0.62-1.43)	.766	0.85 (0.58-1.26)	.422	0.84 (0.55-1.28)	.419

Good oral health behaviour								

Toothbrushing more than 3 times per day	0.69 (0.42-1.12)	.134	0.77 (0.43-1.40)	.391	0.79 (0.47-1.32)	.368	0.99 (0.55-1.79)	.977
Drinking sports drinks frequently§	0.61 (0.34-1.11)	.108	0.79 (0.39-1.61)	.516	1.00 (0.60-1.66)	.992	1.57 (0.84-2.92)	.156
Using dental floss	0.67 (0.47-0.94)	.021	0.88 (0.56-1.40)	.594	0.57 (0.40-0.81)	.002	0.73 (0.47-1.15)	.175
Toothbrushing more than 5 minutes per brushing	0.78 (0.56-1.10)	.157	0.79 (0.54-1.16)	.235	0.77 (0.55-1.07)	.120	0.70 (0.48-1.03)	.074
Visiting dentists regularly more than once a year	0.78 (0.56-1.10)	.157	0.79 (0.49-1.26)	.318	0.82 (0.59-1.14)	.232	1.21 (0.78-1.87)	.398
Using fluoride toothpaste	1.20 (0.86-1.68)	.277	1.61 (1.08-2.42)	.020	0.95 (0.68-1.33)	.774	1.17 (0.80-1.72)	.419
Eating many sweet foods§	1.32 (0.93-1.85)	.117	1.07 (0.68-1.67)	.767	1.33 (0.94-1.87)	.105	1.11 (0.72-1.69)	.638
Experiencing with toothbrushing instruction	0.92 (0.61-1.39)	.694	1.07 (0.69-1.67)	.751	0.73 (0.45-1.18)	.198	0.86 (0.52-1.41)	.551
Using toothpaste	1.12 (0.74-1.68)	.601	1.28 (0.79-2.07)	.309	1.10 (0.72-1.67)	.651	1.16 (0.72-1.87)	.535

⁎ Non periodontal condition = 0, With periodontal condition = 1.

† Confidence interval.

‡ Adjusted odds ratio for sex and each of the variable at baseline.

§ Negative and reverse designed items.

¶ Logistic regression analysis.

## Discussion

This study is the first to investigate the effects of a developed school-based online oral health programme with a periodontal examination results sheet on subjective oral symptoms, oral health knowledge and awareness, and oral health behaviours among high school students with a 1-year follow-up. The results showed that the percentage of students who experienced gingival swelling in the PC group increased significantly after 3 months, and the percentage of students who experienced gingival bleeding in the non-PC group decreased significantly after 3 months; however, these 2 groups did not significantly differ between baseline and 1 year later. In addition, the awareness of gingival swelling and awareness of bleeding at 3 months were positively associated with the presence of periodontal conditions after adjustment for sex. The students may have been made aware of their own periodontal condition thorough their periodontal examination results sheet, and this awareness may have been limited 1 year later. Therefore, the sheet might have contributed to temporary changes in awareness of subjective oral symptoms. Moreover, a previous survey reported that longer toothbrushing time was negatively associated with the awareness of gingival bleeding among Japanese adolescents.^27^ Therefore, the improvement in toothbrushing time in the non-PC group might have contributed to the decrease in the awareness of gingival bleeding.

The percentage of participants’ oral health knowledge improved significantly after 1 year, which suggested that the online health education programme might have been effective in improving oral health knowledge among participants in this study, as did other previous studies conducted on online health education programmes.^18^^,^^19^ According to the analysis by group, as the percentage of participants’ interest in oral health in only the PC group increased significantly, their awareness of having a periodontal condition according to the questionnaire might have affected the increase.

The students’ oral health behaviours, such as drinking sports drinks frequently, brushing their teeth more than 5 minutes each time, and using fluoride toothpaste, improved significantly after 3 months and 1 year. The instructions were included in the online education programmes and described on the sheet. Therefore, these instructions might have contributed to the improvement in oral health behaviours. The toothbrushing instructions provided by a dental hygienist were delivered as live videos through an online education programme, which might have contributed to the improvement in their experience with toothbrushing instruction.

According to the analysis by group, oral health using fluoride toothpaste significantly improved in only the PC group. The importance of these behaviours was included in the online education programme. In addition, a warning against drinking sports drinks frequently was described in the sheet, as this is positively associated with severe gingivitis and dental caries.^28^^,^^29^ Therefore, these instruction programmes might have contributed to the improvement in their behaviours.

A previous study conducted at the same school reported that less time spent brushing teeth and drinking sports drinks more frequently were positively associated with having a periodontal condition.^30^ Therefore, these improvements in oral health behaviours might contribute to improving oral hygiene and preventing periodontal diseases, although further studies are needed to determine the effect.

Total of 27.6% of the participants used dental floss at baseline, which was similar to the percentage of adolescents aged 15 to 19 years in the national survey (25.6%).^21^ The percentage did not increase significantly after the programme was implemented, although the importance and methods of use of the programme were explained in the oral health education programme. A previous study conducted in a Japanese high school reported that using dental floss was negatively associated with the presence of dental calculus and a pocket depth of 4 mm or more.^26^ A study in Hong Kong reported the association of periodontal disease with floss use in young adults.^30^ In addition, a systematic review concluded that interdental cleaning devices were effective at reducing gingivitis and plaque.^31^ Therefore, flossing is important for preventing gingivitis in adolescents, and further improvements in oral health care programmes are needed to increase its use.

The percentage of students who visited dentists regularly also did not increase significantly after 1 year. The benefit of visiting dentists regularly was explained in the videos and on the sheet. Previous studies in Japan^32^ and other countries^33^, ^34^, ^35^ have shown that screening students at a dental examination or sending a letter for dental attendance to students was not effective at improving dental attendance. Therefore, screenings and school-based oral health education might be difficult for high school students in Japan to improve dental attendance, and a community-based approach may be needed to encourage students to visit dentists regularly.

The levels of good knowledge and attitudes regarding oral health and good oral health behaviour 3 months and 1 year later were higher than those at baseline, which might indicate the effectiveness of the programmes. However, only the use of fluoride toothpaste 3 months later was positively associated with the presence of periodontal conditions. Therefore, it is suggested that improved awareness of periodontal conditions through the use of a periodontal examination results sheet might have a limited effect on improving knowledge, attitudes, and behaviours regarding oral health.

This study has several limitations. First, only 1 high school was surveyed in this study, which may not be generalisable. However, in terms of the use of dental floss and visiting dentists regularly, there were no large differences between the percentages of participants at baseline in this study (27.6% and 49.8%) and those aged 15 to 19 years in a national survey (25.6% and 61.4%, respectively).^21^ Therefore, we believe that this is not a special high school in terms of oral health behaviours.

Second, although some students did not participate in the periodontal examination, distance learning, or questionnaire survey, the participation rates were high (more than 74%) for all the programmes. However, the nonparticipants in the examinations or programmes might have declined participation for various reasons, such as fear of exposing poor oral hygiene, low self-esteem, or a perception of good oral health and lack of interest in participating in the study. A follow-up survey using a questionnaire to determine the reasons for nonparticipation and to improve participation rates may allow for a more accurate evaluation of the effectiveness of these study programmes.

Third, the control groups that receive neither the educational programme nor the clinical examination, or only one of the 2, were not included in this study. The online oral health education programme in this study needed to be administered simultaneously to all students to reduce the cost and implementation burdens of the high school. The participants might have learned about oral health in a school health class or received oral health education in a private dental clinic during the study period, which might have contributed to the improvement in their subjective oral symptoms, knowledge and attitudes regarding oral health, and oral health behaviour. Therefore, to eliminate these confounding factors, further studies should be conducted with a control group of students from schools with similar characteristics.

Fourth, periodontal examination using the PCPI aims to accurately evaluate periodontal disease conditions in a study. However, the Plaque Index,^36^ which enables the evaluation of oral hygiene conditions, might better reflect attitudes towards the practice of oral hygiene regimens than the PCPI. Therefore, to further enhance educational effectiveness, this examination should be replaced with an appropriate examination, such as the Plaque Index, and the results should be reflected in the examination results sheet.

Fifth, the recommended brushing time is usually 2 minutes in America and 3 minutes in Europe.^37^ A previous cross-sectional study conducted at the same school as this study reported that more than 5 minutes or more of toothbrushing was positively associated with having no calculus.^26^ Moreover, a study regarding the association between gingival bleeding and the duration of toothbrushing among 9098 Japanese university students showed that for students who brushed their teeth for “1 minute or less” or “2-3 minutes”, the risk of having gingival bleeding was 1.57 (95% CI: 1.39-1.78) and 1.26 (1.14-1.39) times greater than for those who brushed them for “4 minutes or more”, respectively.^27^ Therefore, toothbrushing for more than 5 minutes was recommended in this programme. Although the percentage of patients who brushed their teeth for more than 5 minutes increased significantly from 47.6% at baseline to 54.8% 1 year later, further studies are needed to investigate the effectiveness of the duration of toothbrushing on the improvement in periodontal condition.

Last, this study was conducted only in Japan. An online oral health education programme can be made available in other countries if the systems are introduced. However, further studies are needed to determine the effectiveness of these programmes in other communities or countries.

## Conclusions

A school-based online oral health education programme with a periodontal examination results sheet was administered to high school students aged 15 to 17 years. The results showed that their oral health knowledge and behaviours, except for the use of dental floss and visiting dentists regularly, improved after the programme. The awareness of gingival swelling and bleeding and the use of fluoride toothpaste at 3 months were positively associated with the presence of periodontal conditions after adjusting for sex and each of the variables at baseline. The online oral health education programme contributed to improving oral health knowledge and behaviours among high school students, and the awareness of periodontal condition according to the periodontal examination results sheet might have improved the awareness of gingival swelling and bleeding at 3 months. However, further studies are needed to investigate the effectiveness of these oral health education programmes in other communities and countries.

## Conflict of interest

None disclosed.

## Appendix Supplementary materials

Image, application 1

Image, application 2

## Footnotes

Supplementary material associated with this article can be found in the online version at doi:10.1016/j.identj.2024.07.002.
