# Supplementary material for: Development of a School-Based Online Periodontal Education Programme for Adolescents
Source: Int Dent J. 2024 Jul 23;75(2):502–13. doi: 10.1016/j.identj.2024.07.002 (PMC11976471; doi:10.1016/j.identj.2024.07.002)
Supplement: Supplementary file 1 [file mmc1.docx]

| Year　　　 Class　　　　　 Name  Your periodontal (gum) condition | | | | | 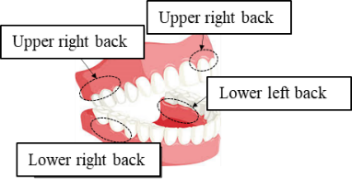 | | |
| --- | --- | --- | --- | --- | --- | --- | --- |
| Upper right back | | Upper anterior | | Upper left back | | | |
| Bleeding | Presence/Absence | Bleeding | Presence/Absence | Bleeding | | Presence/Absence | |
| Tartar | Presence/Absence | Tartar | Presence/Absence | Tartar | | Presence/Absence | |
| Swollen gum | Moderate/  Severe/Absence | Swollen gum | Moderate/  Severe/Absence | Swollen gum | | Moderate/  Severe/Absence | |
| Lower right back | | Lower anterior | | Lower left back | | | |
| Bleeding | Presence/Absence | Bleeding | Presence/Absence | Bleeding | | Presence/Absence | |
| Tartar | Presence/Absence | Tartar | Presence/Absence | Tartar | | Presence/Absence | |
| Swollen gum | Moderate/  Severe/Absence | Swollen gum | Moderate/  Severe/Absence | Swollen gum | | Moderate/  Severe/Absence | |
| What are gingivitis and tartar? | | | | | | | |
| Gingivitis is a condition in which the gums are inflamed. When gingivitis develops, the gums become red and swollen and bleed at the slightest external stimulus. As gingivitis progresses, the inflammation spreads to deeper tissues supporting the teeth (periodontitis), and in the worst case, tooth loss may occur (see figure on the right). | | | | | |  | |
| The main causes of gum disease are dental plaque and tartar. Insufficient tooth brushing and excessive sugar intake cause plaque to accumulate in gingival pockets between the teeth and gums, leading to inflammation. It is said that there are more than 1 billion bacteria in 1 mg of plaque, and inflammation of the gums is caused by these bacteria. Tartar (right photo) is a hardened form of plaque that cannot be removed by brushing, and you need to visit a dentist to remove this. | | | | | |  Tartar | |
| What are the systemic effects of gum disease? | | | | | | | |
| Plaque burrows into periodontal gum pockets and progressively destroys periodontal tissue, and toxic substances released by inflammation enter the system through the blood vessels of the gums, causing or aggravating various diseases. Inflammatory substances are also involved in dementia, stroke, arteriosclerosis of blood vessels, diabetes, premature and low birth weight births, etc. (see figure on the right). | | | | | | |  |
| How to prevent gum disease? | | | | | | | |
| - Proper daily brushing is important to remove plaque that causes gum disease. - Brushing your teeth for at least 5 minutes after each meal. - Using dental floss to remove plaque between teeth. - Not drinking sugary drinks too often (e.g., sports drinks), as they can cause tooth decay and gingivitis. | | | | | | | |
| Benefits of regular dental visits | | | | | | | |
| - Best way to keep your mouth healthy and prevent bad breath. - Only way to remove tartar which causes gum disease. - Early detection of carious teeth and gum disease and treatment before the pain becomes severe. - Dental hygienist will instruct you how to brush teeth well, clean your mouth daily, and prevent bad breath. | | | | | | | |

Supplementary Figure 1 Periodontal examination results sheet


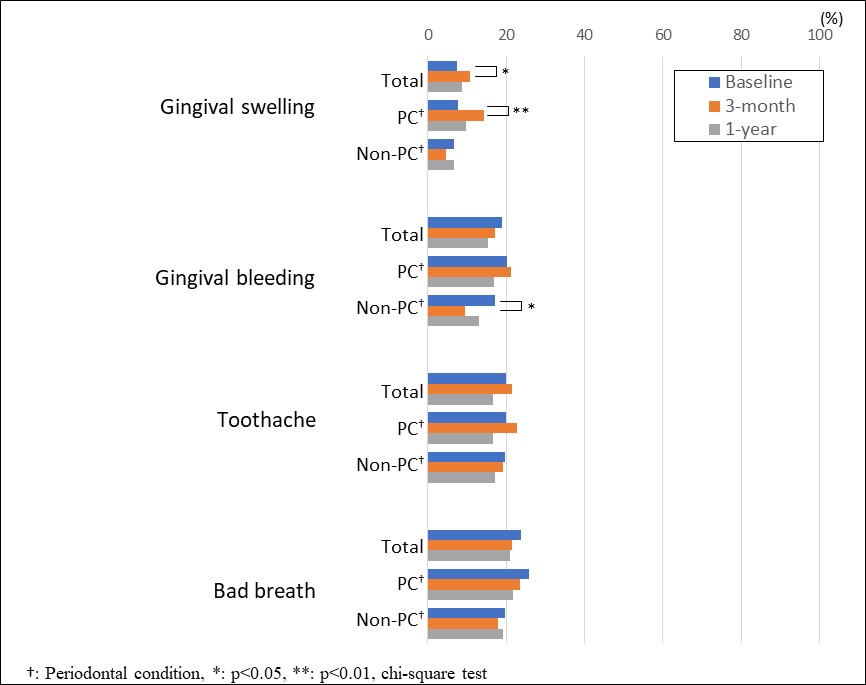


Supplemental Figure 2 Changes in subjective oral symptoms in the PC^†^ and non-PC^†^ groups


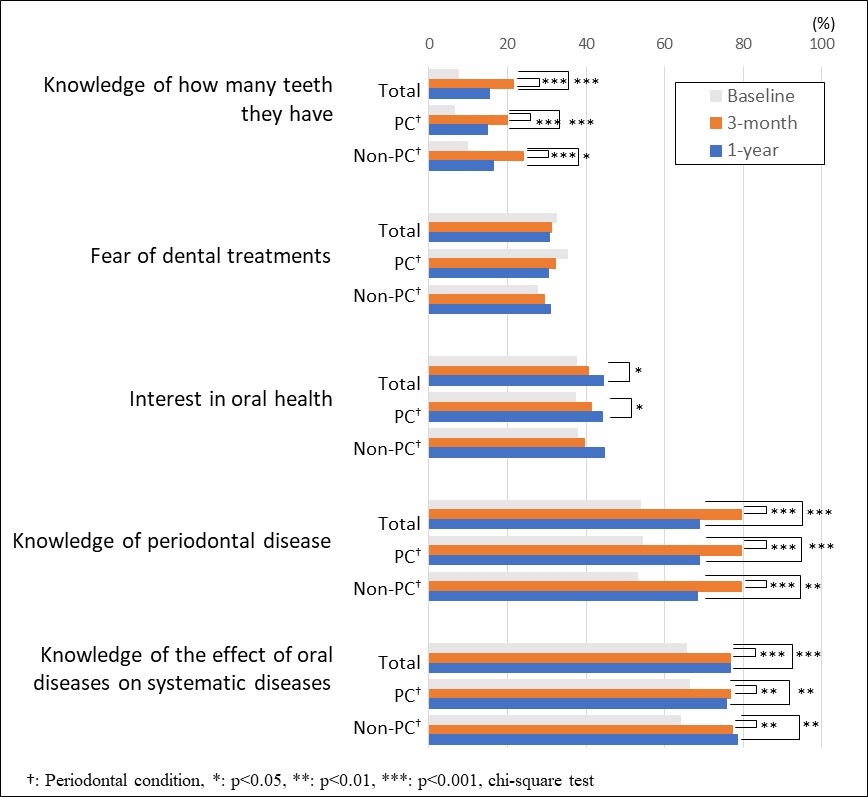


Supplementary Figure 3 Changes in knowledge and attitudes regarding oral health in the PC^†^ and non-PC^†^ groups


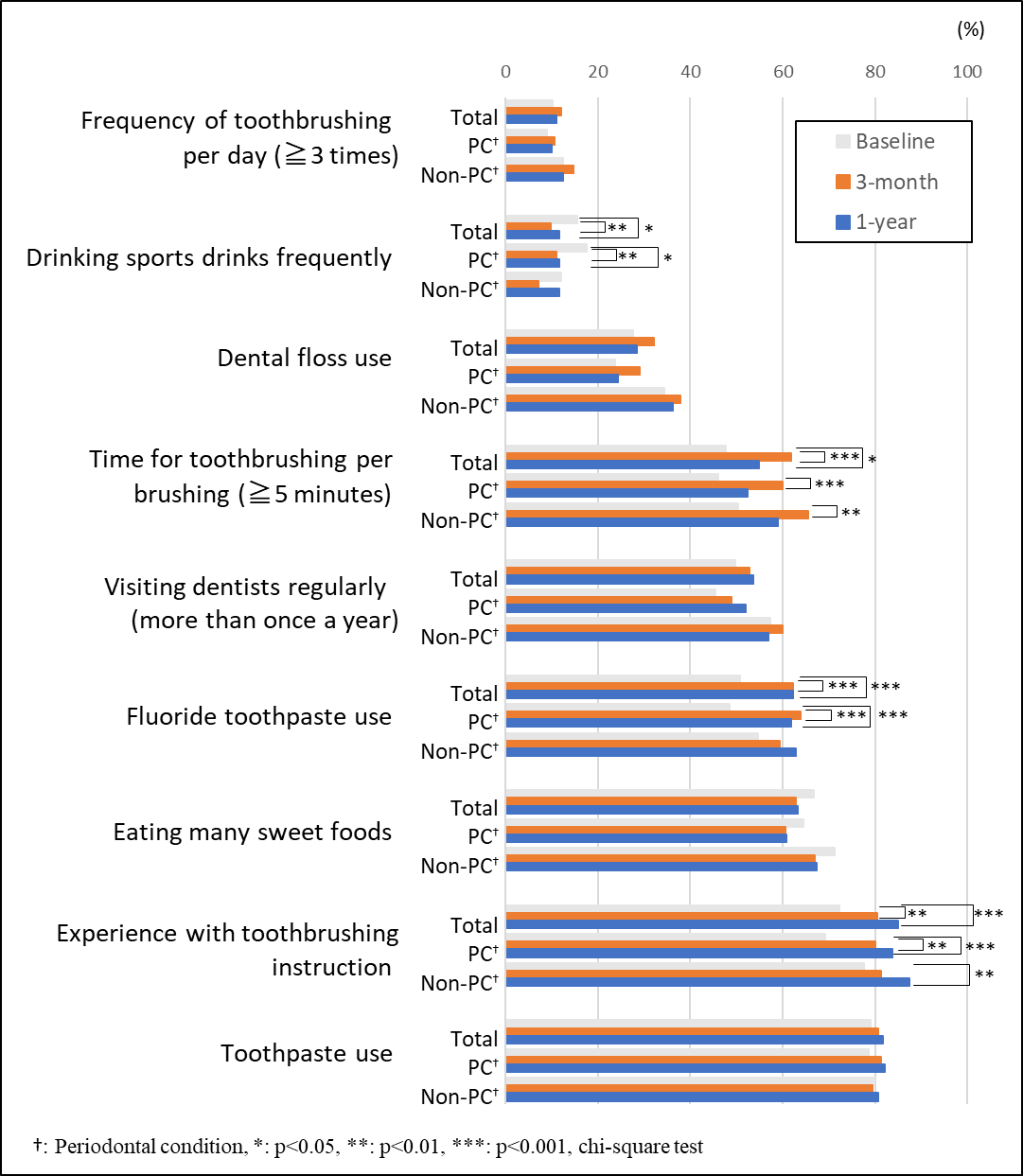


Supplemental Figure 4 Changes in oral health behaviour in the PC^†^ and non-PC^†^ groups
