# Supplementary material for: Development of a School-Based Online Periodontal Education Programme for Adolescents
Source: Int Dent J. 2024 Jul 23;75(2):502–13. doi: 10.1016/j.identj.2024.07.002 (PMC11976471; doi:10.1016/j.identj.2024.07.002)
Supplement: Supplementary file 2 [file mmc2.docx]

| Supplementary Table 1 Questionnaire used in this study | | |
| --- | --- | --- |
|  | Question | Answer |
| Oral symptom | |  |
| Q1 | Do you have a toothache when you chew something? | Yes/No |
| Q2 | Do you bleed when you brush your teeth? | Yes/No |
| Q3 | Are your gums swollen? | Yes/No |
| Q4 | Are you concerned about bad breath? | Yes/No |
| Knowledge and attitudes regarding oral health | |  |
| Q5 | Do you know how many teeth you have? | Yes/No |
| Q6 | Do you know about periodontal disease? | Yes/No |
| Q7 | Do you think oral diseases affect systemic diseases? | Yes/No |
| Q8 | Do you fear dental treatments? | Yes/No |
| Q9 | Are you interested in oral health? | Yes/No |
| Oral health behaviour | |  |
| Q10 | How many times a day do you brush your teeth? | 3 times or more  2 times  1 time or less |
| Q11 | How long do you brush your teeth for each brushing? | ≧5 minutes  3-5 minutes  <3 minutes |
| Q12 | Do you use dental floss? | Yes/No |
| Q13 | Do you eat many sweet foods? | Yes/No |
| Q14 | Do you use toothpaste? | Yes/No |
| Q15 | Do you use fluoride toothpaste? | Yes/No |
| Q16 | Have you had any experience with tooth brushing instruction? | Yes/No |
| Q17 | Do you frequently drink sports drinks? | Yes/No |
| Q18 | Do you visit the dentist regularly? | Yes/No |

| Supplementary Table 2 Contents of online oral health education programmes | | |
| --- | --- | --- |
| Date  (Time) | Form | Contents (Time period) |
| 2022/12/13 | Video | Chapter 1: Sports Dentistry (15 minutes) |
| (10:50-12:10) |  | Tooth and jaw structure |
|  |  | Tooth trauma and tooth dislocation |
|  |  | Mouthguard |
|  |  | Risks of sports drink |
|  |  | Mouth breathing |
|  |  | Bad breath |
|  |  | Importance of chewing |
|  | Live video | Video commentary by a dental hygienist on chapter 1 (15 minutes) |
|  | Video | Chapter 2: Introduction about oral health professionals (20 minutes) |
|  |  | Introduction to the work of four dental hygienists |
|  |  | Introduction to the work of one dental technician |
|  | Live video | Video commentary by a dental hygienist on chapter 2 (5 minutes) |
| 2023/1/16  (10:50-12:10) |  | Periodontal disease examination result notification form and a toothbrush were distributed to each student |
|  | Live video | Chapter 3: Periodontal disease (16 minutes) |
|  |  | Oral bacteria and plaque |
|  |  | Microscopy images of oral bacteria |
|  |  | Effect of plaque on caries and periodontal disease |
|  |  | What is periodontal disease? |
|  |  | Symptoms of periodontal disease |
|  |  | Progression of periodontal disease |
|  |  | Effect of oral diseases on systematic diseases |
|  | Live video | Video commentary on chapter 3 and explanation how to read by a dental hygienist (10 minutes) |
|  | Video | Chapter 4: Plaque control (15 minutes) |
|  |  | How to brush teeth |
|  |  | How to use dental floss |
|  |  | Benefit of toothpaste including fluoride toothpaste |
|  |  | Benefit of regular dental visits |
|  |  | Calculus removal treatment at dental clinic |
|  | Live video | Toothbrushing instruction by a dental hygienist |
